# Study protocol: Optimising patient positioning for the planning of accelerated partial breast radiotherapy for the integrated magnetic resonance linear accelerator: OPRAH MRL

**DOI:** 10.1186/s13014-024-02517-3

**Published:** 2024-09-17

**Authors:** Jenna Dean, Nigel Anderson, Georgia K. B. Halkett, Jessica Lye, Mark Tacey, Farshad Foroudi, Michael Chao, Caroline Wright

**Affiliations:** 1 Radiation Oncology, Olivia Newton John Cancer Wellness and Research Centre, Austin Health, PO Box 5555, Heidelberg, VIC 3084 Australia; 2 Department of Medical Imaging and Radiation Sciences, Faculty of Medicine, Nursing and Health Sciences, Monash University, Wellington Rd, Clayton, VIC 3800 Australia; 3 Curtin School of Nursing/Curtin Health Innovation Research Institute, Faculty of Health Sciences, Curtin University, GPO Box U1987, Perth, WA 6845 Australia; 4 School of Health and Biomedical Science, RMIT University, 124 La Trobe St, Melbourne, VIC 3000 Australia

**Keywords:** Early breast cancer, Magnetic resonance Linac, Radiotherapy, Accelerated partial breast irradiation, Patient positioning, Prone, Supine

## Abstract

**Background:**

Accelerated partial breast irradiation (APBI) is an accepted treatment option for early breast cancer. Treatment delivered on the Magnetic Resonance integrated Linear Accelerator (MRL) provides the added assurance of improved soft tissue visibility, important in the delivery of APBI. This technique can be delivered in both the supine and prone positions, however current literature suggests that prone treatment on the MRL is infeasible due to physical limitations with bore size. This study aims to investigate the feasibility of positioning patients on a custom designed prone breast board compared with supine positioning on a personalised vacuum bag. Geometric distortion, the relative position of Organs at Risk (OAR) to the tumour bed and breathing motion (intrafraction motion) will be compared between the supine and prone positions. The study will also investigate the positional impact on dosimetry, patient experience, and position preference.

**Methods:**

Up to 30 patients will be recruited over a 12-month period for participation in this Human Research Ethics Committee approved exploratory cohort study. Patients will be scanned on the magnetic resonance imaging (MRI) Simulator in both the supine and prone positions as per current standard of care for APBI simulation. Supine and prone positioning comparisons will all be assessed on de-identified MRI image pairs, acquired using appropriate software. Patient experience will be explored through completion of a short, anonymous electronic survey. Descriptive statistics will be used for reporting of results with categorical, parametric/non-parametric tests applied (data format dependent). Survey results will be interpreted by comparison of percentage frequencies across the Likert scales. Thematic content analysis will be used to interpret qualitative data from the open-ended survey questions.

**Discussion:**

The results of this study will be used to assess the feasibility of treating patients with APBI in the prone position on a custom designed board on the MRL. It may also be used to assist with identification of patients who would benefit from this position over supine without the need to perform both scans. Patient experience and technical considerations will be utilised to develop a tool to assist in this process.

*Trial registration* Australian New Zealand Clinical Trials Registry (ANZCTR): ACTRN1262400067583. Registered 28th of May 2024. https://www.anzctr.org.au/ACTRN12624000679583.aspx

## Background

Worldwide, breast cancer is now the most commonly diagnosed form of cancer in women [1]. Early breast cancer (an invasive breast cancer, contained within the breast which may, or may not have spread to the lymph nodes in the breast or axilla) is the most common presentation of breast cancer in Australia [1]. Most patients with early breast cancer receive breast conservation surgery and radiotherapy, with studies demonstrating the highest breast cancer specific survival in this group at 98.8% [2]. It has been demonstrated that the perceived burden of radiotherapy (access, cost, travel, time away from home or off work—typically 3–6 weeks) may influence the patient’s decision to access this treatment [3]. Accelerated partial breast irradiation (APBI) is a promising treatment option, minimising radiation exposure to normal tissue and reducing toxicity, without compromising cancer control [4, 5]. In addition, ABPI is more convenient for patients (5 treatments vs. conventional 15–25 treatments) allowing for scarce radiation resources to be utilised more efficiently [6].

Recurrences in early breast cancer are most likely to occur close to the position of the original tumour, APBI focusses treatment to the tumour bed with a small margin to encompass the area at highest risk of recurrence [4]. To deliver this treatment safely and accurately, it is imperative that clinicians can access state of the art imaging and treatment technology. The superior soft tissue contrast provided by Magnetic Resonance (MR) imaging when compared to standard conventional cone beam computed tomography (CBCT) provides enhanced visual acuity of both the target and adjacent healthy tissue [7]. The integrated MR Linear Accelerator (MRL) facilitates daily plan adaption to account for variations in anatomy or positioning, as well as granting real time positional information during treatment delivery, ensuring dose is delivered to the intended target.

**Fig. 1:**
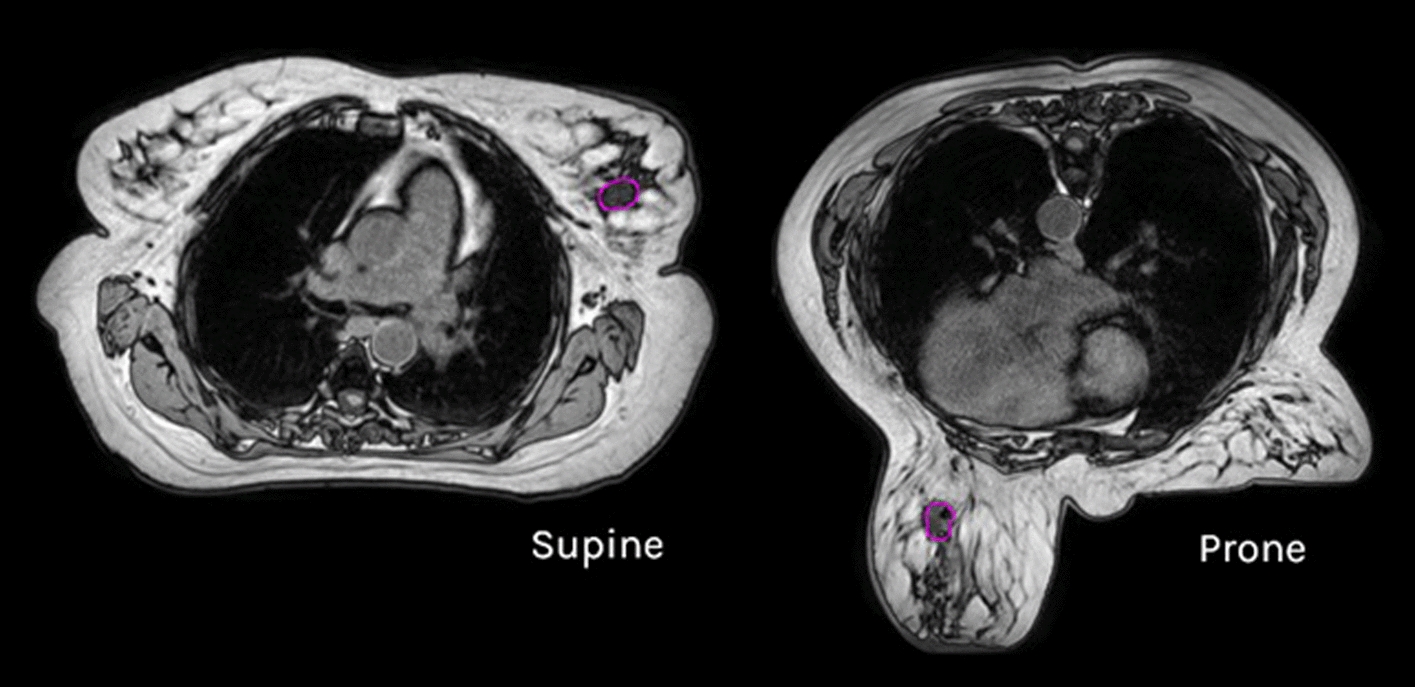
Visualisation of change in position of anatomy on T1 MR Simulation images

The effects of geometric distortion, patient, and breathing motion as well as physical limitations of the bore size and field of view are known in breast MR imaging. These translate with additional considerations to treatments using the integrated MRL [8]. Numerous studies have considered supine versus prone positions in different cohorts of patients for the delivery of whole breast [9, 10] and APBI [11]. They have suggested that a number of patients (mainly those with larger, pendulous breasts) would benefit from treatment in the prone position citing lower doses to Organs at Risk (OAR), particularly the ipsilateral lung [12]. The positional change in relative anatomy between supine and prone supports the decrease in lung dose and improved dosimetry as the position moves the target (tumour bed) away from the chest wall, avoiding lateral breast fall as can be seen in Fig. 1. Some studies have cited slightly higher heart doses in the prone position for whole breast radiotherapy, but this is not consistent across the literature and may be more dependent on the location of the tumour in the context of APBI [9, 10].

Few studies have considered the comparison of positions for individual patients for MRL APBI treatment, with some groups suggesting that prone treatment is not feasible on this machine [8]. Until recently, commercially available prone breast immobilisation devices were neither MR safe nor practical for the limited space in the bore. Our team, therefore, worked with an industry partner (CDR Systems Inc., Calgary, Canada) to develop a MR safe solution. Therefore, we plan to assess whether the purpose-built prone board will provide a solution to support the treatment of APBI patients on the MRL and thoroughly explore the considerations of delivering this type of adaptive treatment in a magnetic environment.

## Methods/design

### Aims

The primary aim of this study is to investigate the benefits and limitations of supine and prone treatment positions for APBI planned for the MRL. This will involve a comparison of the location of the tumour bed and its proximity to OAR in each position, an assessment of the difference in geometric image distortion close to the target, and measurement of the of the patient’s breathing motion.

We hypothesise that prone positioning for APBI will result in less geometric distortion and less breathing (intrafraction) motion when compared to supine positioning. It will also increase the relative distance between the target volume and organs at risk (heart, lung(s), and chest wall) resulting in lower doses to these structures.

Primary outcomes:

1. Quantification of geometric image distortion on planning MRI scans in both the supine and prone positions to determine whether there is a position specific difference.
2. Measurement of relative distance from the Planning Target Volume (PTV) to the OAR in the supine and prone positions.
3. Assessment of breathing motion for each patient in each position to determine whether motion is larger in the supine or prone position.

Secondary aims of this study include:

1. To assess the differences in dose to the target, organs at risk and normal tissue for each patient between the supine and prone positions on planning MRI scans (simple synthetic computed tomography scans (CTs) generated from anatomical contours with bulk density overrides).
2. To explore the patient experience of having an MRI simulation scan and whether there is a preference for the supine or prone position.

Secondary outcomes:

1. Assess differences in dosimetry (dose in Gray) to the target, OAR and normal tissue for each patient between the supine and prone positions on synthetic CTs generated from the MRI scans.
2. Analyse survey responses (content analysis) to ascertain whether there is a patient preference for the supine or prone position.

### Study design

This is a prospective, exploratory single-centre pilot study of patients undergoing APBI for early-breast cancer. Patients meeting the American Society for Radiation Oncology (ASTRO) favourable and certain cautionary criteria for APBI will be invited to participate (Table 1) Patients with known contraindications to radiotherapy such as ataxia telangiectasia and systemic sclerosis will be excluded [13]. Participants will be asked to provide written informed consent. Participation will not alter the management of the patients but will inform the decision-making process for the choice of treatment position for future patients treated with ABPI, based on the study outcomes.

**Table 1:** ASTRO APBI Favourable and cautionary suitability criteria [13]

Features	
Age	≥ 50 years
Subtype	IDC and DCIS
Size	≤ 3cm
Grade	< 3 (1 or 2)
Margin	Negative ≥ 2 mm
Lymphovascular space invasion	Absent
Hormone (ER) Positivity	Yes
Her2	Negative
Nodal Status	Negative or pN_ITC_

Participants will be scanned on a dedicated Philips Ingenia Ambition MR-RT 1.5T MRI simulator (Koninlijke Philips N.V., Amsterdam, Netherlands) in both the supine and prone positions for APBI as per current departmental standard of care. Assessment of positioning, geometric distortion, patient and breathing motion, and dosimetry will be completed using the de-identified data from these scans. In addition, immediately following the MRI simulation session, participants will be invited to complete a short anonymous electronic survey (using closed and open-ended questions) about their experience of the planning MRI scans (MR specific sensations, information provided about the procedure and positional preferences). CT simulation and treatment will be completed as per standard protocol with appropriate immobilisation equipment in the preferred position (supine or prone as a clinical decision after MR image review) with no further study-based requirements.

### Recruitment

Participants will be invited to participate and provided with the relevant study information to make an informed decision about their participation by their Radiation Oncologist (RO) in their initial consultation if they are eligible for treatment with APBI and meet the study inclusion criteria.

### Positioning assessment

Participants will attend for MRI simulation prior to CT simulation in alignment with standard departmental protocol. For the supine MRI scan, patients will be positioned with both arms up supported by a custom vacuum bag. For the prone MRI scan, patients will be positioned on the Low Procline™ (CDR Systems Inc. Calgary, Canada), which was developed in collaboration with one of our investigators. To make the positional, distortion and breathing motion assessment the standard breast MRI sequences (T1, T2 and Cine imaging) will be acquired in each position, optimised for each laterality. The minimum distance will be calculated between the Target volume and heart, lung(s), skin rind (5mm inside the patient contour), and chest wall.

Participants will be assigned to have either the supine or prone MRI scan first (alternating between the positions) as they are recruited, to reduce bias towards the first or second scan. The order will also be recorded and considered in the analysis of results.

### Geometric distortion assessment

Geometric distortion in MRI is a known artifact arising from inherent magnetic field inhomogeneities that becomes larger further from the imaging isocentre [14]. Geometric distortion will be assessed in MIM Maestro® version 7.1 or later (MIM) utilising the dPhantom (3DOne Australia Pty Ltd, QLD, Australia), a specific MRI distortion phantom containing a 2 cm MRI visible grid. Two MR images will be taken of the phantom; a reference that is centred in the bore, and a second MR will be taken with the phantom shifted a known distance to cover the region of interest, e.g., Breast. Patient images in the supine and prone positions will be contoured, and structures transferred to the shifted MR. The shift from the ground truth 2 cm grid will be calculated within each contour. The mean and maximum shifts will then be calculated as a measure of geometric distortion for each contour to support comparison of distortion between the two positions.

### Breathing motion assessment

Cine imaging sequences in sagittal-coronal directions acquired during the MRI simulation in both the supine and prone positions will be used to assess the range of breathing (intrafraction) motion. Images will be collected for 1-min intervals at multiple timepoints within the session in each position. Imaging and treatment will be performed with the patient free breathing. Motion will be measured across a minimum of five breathing cycles using tumour or surrogate edge displacement in Anterior/Posterior, Superior/Inferior and Left/Right directions. The exhale baseline drift will be assessed across the breathing phases and the mid-point between maximum and minimum exhale position will be used as the reference point. The maximum and average extents of the motion will be measured in both the supine and prone positions for each patient.

### Dosimetric assessment

Patients will have all subsequent planning and treatment performed as per the standard departmental protocol. There is no change to usual care for radiotherapy planning procedures.

For study purposes, each patient’s supine and prone MR scans will be contoured (target volumes, appropriate OAR and structures requiring a specific electron density override) using MIM then used to make a pseudo-CT; average densities will be acquired from the collective supine and prone CT scans (acquired as part of the patient’s standard of care) and applied as average population bulk densities to structures as appropriate in the Elekta Monaco™ version 5.51.11 or later (Elekta solutions AB, Stockholm, Sweden) radiotherapy treatment planning system.

In this study protocol, a MR-only workflow will not be used. Every patient will receive a CT scan following their MR sim appointment in the treatment position which will provide patient specific electron densities as per current clinical practice in our department. Contours will be applied to the MRI and bulk electron densities will be assigned to the contours in the planning system as per the current MRL workflow to create the synthetic CT. Quality assurance of the average population electron density will be performed in line with the departmental protocol for online adaptive treatment, recalculating the treatment plan on the CT and comparing the dosimetry from the planning CT and the synthetic CT.

Target volumes will be contoured by RO study investigators and all other structures by RTs. The primary target volume will consist of visible seroma and the surrounding post operative tumour bed. Standard departmental margins will be applied to generate the Clinical Target Volume (CTV) and planning target volume (PTV). APBI plans for 30 Gray (Gy) in 5 fractions with the Elekta Unity™ (Elekta solutions AB, Stockholm, Sweden) MR Linac beam model will be completed for comparison on the pseudo-CT scans in both the supine and prone positions. Beam angles will be optimised to the ipsilateral side and adjusted appropriately for tumour location, patient position, treatment laterality and to avoid entry through the cryostat pipe. Planning considerations specific to the Unity; couch top, MR radiofrequency coil and structure/density layering will also be applied. Calculation and sequencing parameters will adhere to standard departmental protocol for APBI. Doses to the targets and OAR (Heart, Lung(s), ipsilateral chest wall, ipsilateral (breast–PTV), contralateral breast and skin rind (5mm)) will be collected to ascertain whether a particular position provides a benefit over the other dosimetrically.

### Patient experience survey

Immediately following the simulation MR scans, participants will be asked to complete a simple, short anonymous survey to assess their experience of each position (see Appendix 1 “Patient Experience Survey”). The survey will ask patients to rate their experience of each of the positions and other common experiences of MRI scans on a Likert scale with an opportunity to explain their rating as free text comments.

This survey has been adapted and developed specifically for this study from a similar tool used by Barnes et al. [15] to investigate the patient experience of treatment on the MRL, as no validated tools were identified in the literature. The survey for this study was critically reviewed by four consumers with radiotherapy treatment experience prior to protocol submission for ethical approval. The survey will be made available to participants on an electronic device at the time of their simulation appointment.

### Sample size estimation

Recruitment will cease at 12 months or when 30 patients have been recruited. This pragmatic sample size is based on the previous 12-month period of simulation appointments for this diagnosis.

Where available, existing MRI and CT datasets collected as standard of care for APBI patients will be included in the geometric distortion, positioning and dosimetry analysis aspects of the study. It is anticipated that up to an additional 30 datasets will be included in this analysis, with this de-identified data accessed under a Human Research Ethics Committee (HREC) approved waiver of consent.

### Statistical analysis

#### Positioning assessment

To compare the supine and prone positioning, categorical data will be presented as counts and frequencies, with means and standard deviations (for normally distributed data) or median and inter-quartile ranges (for non-normally distributed data) used to present continuous variables. Where appropriate, paired parametric or non-parametric tests will be applied to test for differences or equivalence between the supine and prone positions.

#### Patient experience

Quantitative survey results will be interpreted by comparison of percentage frequencies across the Likert scales. Chi Squared and Fishers Exact tests may be used to test for differences between categories, with McNemar’s test used to compare paired responses between prone and supine positioning.

Qualitative data from the open-ended survey questions will be analysed using thematic content analysis [16].

Data will be collected in a Research Electronic Data Capture (REDCap) database and/or Microsoft Excel with statistical analysis planned to be conducted using Stata version 18.0 (StataCorp, College Station, Texas, USA), with a *p*-value of < 0.05 used to indicate statistical significance.

## Discussion

Access to dedicated MR equipment in a radiation oncology setting, particularly in Australia, is relatively new. Our department is uniquely positioned as the only department in the country with both an MRI simulator and an MRL. Given the scarcity of these resources, it’s important to optimise the utilisation for all patients who would benefit from treatment with this technology. Published research has investigated supine and prone positioning for patients receiving whole breast radiotherapy on conventional linear accelerators (Linacs) with most of these studies utilising CT based simulation imaging for assessment [9, 10, 17]. Some studies with smaller sample sizes [8, 12, 18, 19] have evaluated positional aspects of treatment on the MRL for APBI, but have not explored geometric distortion, breathing motion, positional assessment, dose distribution and the patient experience to encompass the aspects explored in this study.

Treatment and planning for APBI requires consideration of the factors that affect the physical properties of radiotherapy in the presence of a magnetic field. The Electron Return Effect (ERE) and Electron Streaming Effect (ESE) which influence dose to skin/air/tissue interfaces all need to be understood and managed to ensure safe application of this technology [20, 21]. An evaluation of the impact of these phenomena is outside of the scope of the current study but may be examined in future work. ERE and ESE have been included in this discussion for noting as they require understanding in addition to planned dosimetry, geometric distortion, breathing and patient motion in this patient cohort.

Considering the factors that impact treatment delivery in patients receiving APBI may inform the ability to identify patients who would benefit from one position over another without requiring a scan in both positions. Body Mass Index (BMI), breast size (bra/cup), tumour bed size and specific location i.e. involved breast quadrant (upper/lower and inner/outer quadrants) information will be ascertained from this work. This will be used to inform the development of a process to streamline positioning prior to simulation, decreasing the length of simulation appointments and the number of required scans.

Whilst this study will address several relevant aspects in relation to optimising patient positioning for APBI on the MRL, there are limitations to be acknowledged. The geometric distortion phantom available in our department is smaller than a patient. The distortion assessment will be completed on an offset phantom to examine the area of interest around the intended target rather than the whole patient. Comparative planning will be completed on pseudo-CT datasets generated on MR images utilising contours and bulk density overrides. The detail we would see on a conventional CT with more variation in Hounsfield Units and therefore density accuracy may affect resultant plans. For this aspect of the study, the authors believe that the impact is negligible given it reflects the current adaptive workflow on the MRL where daily plan adaption is completed on an MR dataset with appropriate bulk densities applied. The authors also acknowledge that to generate robust synthetic CTs in a MR-only workflow, data collection from varied imaging protocols and field strength MRIs would be required. However, this consideration is outside of the scope of this study as all patients will receive a CT scan to provide patient specific electron densities. The method described in our protocol is for the sole purpose of providing a dataset for comparative planning consistent with the current online MRL workflow. The authors recognise that the cohort for the pilot study is relatively small, this was considered in the study development, however it was determined that from this cohort there would be an opportunity to analyse the initial results and expand the study based on the outcomes.

This research will address gaps in knowledge which will support the safe and effective delivery of APBI on the MRL. It will provide opportunities to investigate radiotherapy planning from MR images, decreasing exposure to ionising radiation. It will also provide the groundwork to facilitate prone treatment in this patient cohort with the custom designed board providing access for more patients. It will also inform the practical aspects associated with the use of high dose stereotactic style treatments, pre- and post-operatively, on the MRL providing benefits to patients and optimising utilisation of scarce radiation resources in our department. The application of ultra hypo fractionated and stereotactic pre and post operative APBI treatments on the MRL will continue to be an area of interest for future directions in this research.

## Data Availability

No datasets were generated or analysed during the current study.

## Appendix 1: Participant experience survey

To help us to better understand your experience of the MRI planning scans for your radiotherapy treatment we would like to ask you some questions. You do not have to answer the questions, particularly if they make you feel uncomfortable.

Please rate the following questions and how they apply to your experience using this scale: **1** Not at all, **2** Slightly, **3** Moderately and **4** Very.

**Table:**

	Not at all	Slightly	Moderately	Very
1. I understood the procedure	1	2	3	4
2. I found the position on my tummy comfortable	1	2	3	4
3. I found the noise in the room easy to tolerate	1	2	3	4
4. I found listening to music during my scans helpful	1	2	3	4
5. I found the position on my back comfortable	1	2	3	4
6. I felt cold during my scans	1	2	3	4
7. I found the light in the room easy to tolerate	1	2	3	4
8. I found it easy to breathe lying on my tummy	1	2	3	4
9. I felt hot during my scans	1	2	3	4
10. I found it easy to stay still lying on my back	1	2	3	4
11. I found the amount of time I spent on my back easy to tolerate	1	2	3	4
12. I felt a tingling sensation during my scan	1	2	3	4
13. I found it easy to get into the position on my tummy	1	2	3	4
14. I felt calm during my scans	1	2	3	4
15. I needed more information before the procedure	1	2	3	4
16. I needed more communication from staff during the scans	1	2	3	4
17. I found it easy to get into the position on my back	1	2	3	4
18. I found it easy to stay still lying on my tummy	1	2	3	4
19.I found it easy to breathe lying on my back	1	2	3	4
20. I found the amount of time I spent on my tummy easy to tolerate	1	2	3	4

## Abbreviations

APBI: Accelerated Partial Breast Irradiation
ASTRO: American Society for Radiation Oncology
BMI: Body Mass Index – a measure that relates bodyweight to height.
CBCT: Cone Beam Computed Tomography
CTs: Computed Tomography scans
CTV: Clinical Target Volume
ERE: Electron Return Effect
ESE: Electron Streaming Effect
Gy: Gray
HREC: Human Research Ethics Committee
Linac: Linear Accelerator
MRI: Magnetic Resonance Imaging
MR: Magnetic Resonance
MR Linac/ MRL: Magnetic Resonance Linac (Integrated MRI and Linear Accelerator)
MIM: MIM Maestro®—Software
OAR: Organ(s) At Risk
PTV: Planning Target Volume
RO: Radiation Oncologist
RT: Radiation Therapist
